# Assessing the Healthfulness of University Food Environments: A Systematic Review of Methods and Tools

**DOI:** 10.3390/nu16101426

**Published:** 2024-05-09

**Authors:** Alicia Anne Dahl, Stacy M. Fandetti, Lilian O. Ademu, Ryan Harris, Elizabeth F. Racine

**Affiliations:** 1 Department of Public Health Sciences, College of Health and Human Services, University of North Carolina at Charlotte, Charlotte, NC 28223, USA; sfandett@charlotte.edu; 2 Institute for Advancing Health Through Agriculture, Texas A&M AgriLife Research Center at El Paso, Texas A&M University, El Paso, TX 79927, USA; 3 J. Murrey Atkins Library, University of North Carolina at Charlotte, Charlotte, NC 28223, USA; rharr103@charlotte.edu; 4 Texas A&M AgriLife Research Center at El Paso, Texas A&M University, El Paso, TX 79927, USA; beth.racine@ag.tamu.edu

**Keywords:** food environment, food supply, food services, access to healthy foods, assessment, measurement, social ecological model, emerging adults, university students

## Abstract

The availability, promotion, and price of healthy foods within the university food environment may impact students’ dietary choices. This systematic review summarizes the tools and methods used to assess the healthfulness of university food environments where many students spend a significant portion of their emerging adulthood. Thirty-six global studies published between 2012 and 2022 were sourced from PubMed (NNLM), Cochrane Library (Wiley), Web of Science (Clarivate), APA PsycInfo (EBSCO), CINHAL Complete (EBSCO), ProQuest Nursing, and Allied Health, following PRISMA 2020 guidelines. Of the included studies, 58% were institutional-level audits, 17% examined individual-level perceptions, and 25% combined both. Most institutional-level audits focused on one aspect of the food environment (e.g., eateries, vending machines). For studies examining multiple spaces within the campus environment (38%), comprehensive assessments were limited, and most studies had to employ a combination of assessment tools. Surveys were most often used to gather individual perceptions about the food environment. The Nutrition Environment Measures Survey (NEMS) was the most commonly used tool across all studies. This review highlights the need for a standardized tool, method, or a “healthy” benchmark for specific use at universities to improve methodological rigor and comparability of findings across institutions.

## 1. Introduction

Emerging adulthood is a transitional life stage that includes increased independence and decision-making surrounding health. For many, one such transition is separating from the family unit to attend college. In 2021, 38% of emerging adults aged 18–24 in the United States were enrolled in college [1]. Thus, attending college may present an initial opportunity for emerging adults to establish their own health behaviors, including making food choices. Students enrolled in college often interact with the broader food system through the campus food environment, which is composed of personal and environmental domains that influence dietary behavior [2]. Personal factors include affordability, convenience, and appeal, while environmental factors include availability, cost, and product promotion [2]. Thus, environmental determinants, such as access to nutritious food, may contribute to certain dietary behaviors among college students.

University food environments are unique organizational spaces catering primarily to an emerging adult population, many of whom reside on campus and rely on the university to provide nutritious food [3,4]. The diversity of consumers, including students, faculty, and visitors, on college campuses adds to the distinctive attributes of a campus food environment. Each university campus is independent, with tailored food service contract vendors or dining operations [5]. Universities often offer several options for procuring food on campus, including dining halls, fast food outlets, convenience stores, vending machines, food trucks, pantries, and catered functions. Additionally, food outlets located off campus but within a specific geographic boundary (e.g., a one-mile radius surrounding the campus center) may be considered a part of the university food environment since students might regularly obtain food from these establishments [6,7,8,9,10].

University food environments have predominantly been described as unhealthy, consisting of abundant energy-dense, nutrient-poor food, and sugary beverages [11,12,13,14,15,16,17,18,19,20,21]. Recently, two systematic reviews examined the impact of campus food environments on students’ dietary behaviors [22,23]. One review found that the campus food environment impacts students’ diets; however, the direction of the effect depends on the availability, accessibility, affordability, and acceptability of nutritious food [22]. Additionally, the magnitude and clinical significance of the impact was not determined [22]. Li et al. (2022) found that the university food environment negatively affects students’ dietary choices. Furthermore, the availability of unhealthy food and the high cost of fruit and vegetables prevented students from selecting healthier options [23].

Conducting a campus food environment assessment provides data that can be used for multiple purposes: as a baseline, tracking changes over time, developing effective interventions, and advocating for environmental and policy changes to support students’ nutritional health [7,13,24,25,26]. Objective assessments of the campus food environment include food service contract reviews [5], observational audits [6,7,8,13,15,27,28], and direct observation [11,29]. The aspects of the food environment measured by these tools vary, but generally, they examine the availability, accessibility, and price of healthy and unhealthy food options. Subjective assessments of the university food environment also provide valuable insight into the campus food environment to determine if the food offered is acceptable to consumers. Focus groups [24,30,31,32], student surveys [33], and photovoice methodology [34] have been used to gather these insights. Due to the varied methods and instruments used to draw conclusions about the healthfulness of campus food environments, comparing the findings from studies is often challenging and inconclusive.

Considering the distinctive nature of university food environments, a review of the assessment measures used in this type of organizational food environment is warranted. The current review explores how university food environment assessments have been conducted (tools and methods used) and how “healthy” has been defined and applied based on assessment results. The findings of this review may help administrators, food service providers, or researchers to select appropriate methods and instruments for assessing their campus food environment. The results can also be used to support the development of policies or programs to create healthier food spaces.

### Theoretical Framework

**Figure 1:**
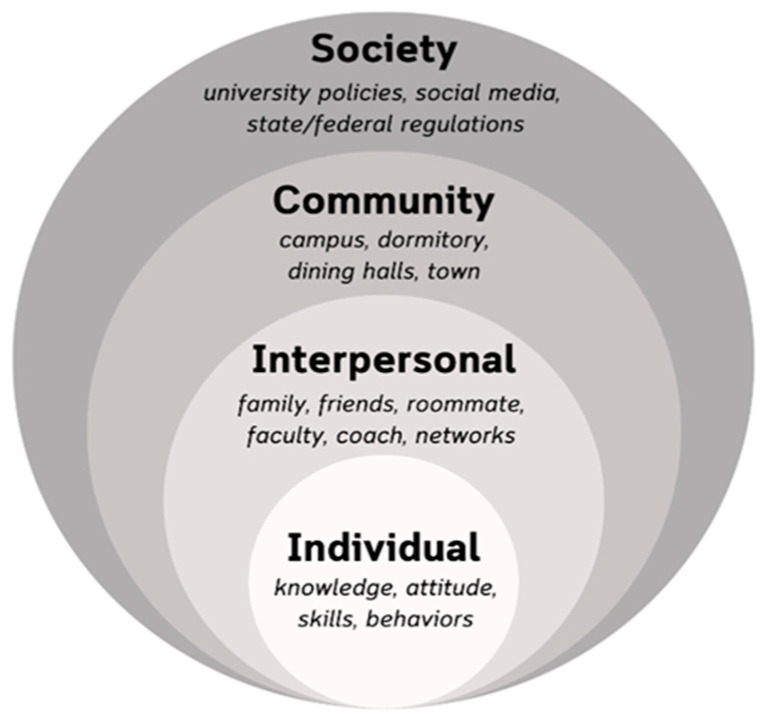
The conceptual framework for this systematic review was informed by the Social Ecological Model to understand four nested levels of influence within the campus food environment. The levels of the model are distinguished by grayscale circles. For example, on an individual level, people hold knowledge, attitudes, skills, and behavioral choices while navigating the food environment. Interpersonal factors such as personal relationships with family, peers, and advisors may influence how an individual responds to the food environment, such as where they dine. Community factors create opportunities or barriers, such as the placement of dining spaces. Lastly, societal factors like campuswide dining policies may determine the types of food offered or meal plans required.

This systematic review used the Social Ecological Model (SEM) to categorize various assessments conducted on university food environments (Figure 1). This framework is essential given the multiple levels of influence on food-related behaviors and the dynamic interplay between individuals and their social contexts [31]. At the individual level, college students may navigate food environments with a set of knowledge, skills, and behavioral preferences. These factors are influenced by the interpersonal social context, such as the behaviors of family members, friends, and other members of their networks. These individual and interpersonal factors are situated within the social context of a broader community, such as the physical campus environment (e.g., the availability of healthy food). Our paper refers to the community level as the institutional setting. Lastly, policies and social norms set at the societal level can influence how college students engage with the campus food environment. For example, a policy requiring first-year students to subscribe to a campus meal plan or live in campus housing would directly influence their accessibility to food and their food-related behaviors. Additionally, the absence of federal regulations on university food environments can directly affect the quality and availability of food on campus. By understanding the scope of the literature through an SEM lens, this comprehensive review is able to identify gaps in the application of food environment assessments within complex university food environments. A complete picture of the dynamic interplay of these processes is critical to improving the healthfulness of university food environments.

## 2. Materials and Methods

This review was conducted in accordance with the Preferred Reporting Items for Systematic Review and Meta-Analysis (PRISMA) 2020 Guidelines for assessing the evidence base [35]. The protocol for the review was registered with the International Prospective Register of Systematic Reviews (PROSPERO; CRD42023398073).

### 2.1. Search Strategy

After consulting the research team, a librarian (RH) with literature-searching expertise identified appropriate concepts and terminology for the review aims. The team then reviewed and revised the search’s list of concepts and terminology. Three primary areas for the search were selected: food environment, food choice and eating behaviors, and the college environment. Each of these concepts encompassed a range of terms. Under the umbrella of the food environment, the included terms were searched but not limited to “meal plans”, “nutrition information”, and “food access”. Within the domain of food choice and eating behaviors, the included terms were searched but not limited to “meal behavior”, “food choice”, and “purchasing behaviors”. Terminology for the college environment search included “university”, “college”, and “post-secondary.” See Supplementary File S1 for a complete list of the search terms.

The librarian conducted initial searches with no restrictions in the following databases: PubMed (NNLM), Cochrane Library (Wiley), Web of Science (Clarivate), APA PsycInfo (EBSCO), CINHAL Complete (EBSCO), ProQuest Nursing, and Allied Health. To supplement the electronic database searches, the research team reviewed the reference list for all included articles and systematic reviews returned with our search results. Depending on the database used, subject term and keyword searching were applied to all searches when appropriate. The librarian initially searched terms in November 2022 for the team to review and revise the search strategy as needed to produce an accurate literature return. All library database searches were completed in January 2023. A research assistant (LA) also searched the literature on Google Scholar by reviewing the first 10 pages of results yielded from the search. The primary terminologies included in the search were “campus food environment” and “assessment”. The Google Scholar search was completed in July 2023, and the results were exported into Covidence as a supplementary search.

### 2.2. Inclusion and Exclusion Criteria

The review’s inclusion criteria aimed to identify studies assessing at least one aspect of the university food environment (e.g., eateries, food stores, dining halls, and vending machines) that reported original research findings. There were no restrictions on the type of study included, whether observational, cross-sectional, validation, reliability, mixed methods, qualitative, or other pre-post studies. Only studies published in English between 2012 and 2022 were included in the review, and there were no restrictions on the country of origin. The primary outcome considered for inclusion was the published results of a campus food environment assessment (e.g., not individual behaviors, such as self-reported dietary patterns). Secondary outcomes were the guidelines or standards used to define “healthy” within these assessments (e.g., federal guidelines) and any policy recommendations provided as part of the publication (e.g., added sugar limits on vending machine products). Studies that did not have a nutritional focus were excluded from the review.

### 2.3. Study Selection

The literature review team included authors AD, SF, and LA. Two reviewers independently screened the titles and abstracts in Covidence [36] to determine eligibility, and a third author resolved any conflicts that arose during the screening process. Next, the articles that met the inclusion criteria were downloaded into Covidence for full-text review. Then, all reviewing authors independently reviewed six articles and discussed the rationale for inclusion or exclusion before moving through the full-text review process. Once the full-text review was completed by two authors, a third author made a determination regarding inclusion or exclusion when necessary. None of the authors were blinded to the journal titles, study authors, or institutions.

### 2.4. Rationale for Categorizing Studies (Institutional vs. Individual-Level Articles)

In our initial search process, we retrieved many studies that used mixed methods to understand the relationship between the food environment and individual behaviors or assessed multiple levels of the food environment. In contrast, others strictly examined the institutional availability of healthy food options on campus through an audit tool. This systematic review used the SEM as a guide to categorize studies by examining the institutional level, individual level, or multiple levels of the campus food environment [31]. Institutional-level studies assessed the campus food environment using objective tools such as a detailed audit instrument, checklist, direct observation, photographs, or other objective measures. Individual-level articles explored perceptions of the campus food environment by collecting data from students or university employees. Articles containing institutional and individual-level elements (e.g., audit tool and student survey) were categorized as multiple SEM levels.

### 2.5. Data Extraction and Analysis

Two reviewers extracted the following information into a spreadsheet: article title, DOI, author(s), year, geographic location, subjects included, setting, assessment type, study design, sample size, sample type, sample characteristics, duration, study objective, primary outcome, a secondary outcome, type of assessment, benchmark criteria for ‘healthy’, and summary of findings. A third review author confirmed the extracted data, and adjustments or updates to the data extraction process were discussed and implemented as necessary. Additionally, two reviewers independently extracted reliability and validity data for each study, as available. Two reviews also independently recorded what was measured by each tool: availability, price, food quality, facilitators of healthy eating, barriers to healthy eating, convenience/access, nutritional information, food promotion, and sustainable practices. Finally, two reviewers documented the healthfulness of food measured within each study (e.g., healthy, unhealthy, or both). When the two reviewers completed the extraction process for tool quality and data description, they met to reach a consensus.

### 2.6. Quality Assessment of Included Studies

The Quality Assessment for Diverse Studies (QuADS) tool was used to assess the methodological quality of the included studies (ranked on a scale of 0–3) in each of the following areas: a theoretical or conceptual underpinning to the research, a statement of research aim/s, a clear description of the research setting and target population, the study design is appropriate to address the stated research aim/s, appropriate sampling to address the research aim/s, the rationale for the choice of data collection tool/s, the format, and content of data collection tool is suitable to address the stated research aim/s, description of data collection procedure, recruitment data provided, justification for the analytic method selected, the method of analysis was appropriate to answer the research aim/s, evidence that the research stakeholder was considered in research design or conduct, and strengths and limitations critically discussed [37]. Two reviewers independently conducted quality assessments, and discrepancies were discussed among the team until a consensus was reached. A quality score for each article was calculated by dividing the individual score (the sum of scores for each of the 13 QuADS items) by the total possible score. For example, if a study had an individual score of 28 out of a possible score of 39 (13 items with a maximum score of 3 each), the quality score was 72%. There is no published guideline for interpreting QuADS results; however, a score allows for the comparison of the methodological and reporting quality of the studies included in this review [37].

## 3. Results

**Figure 2:**
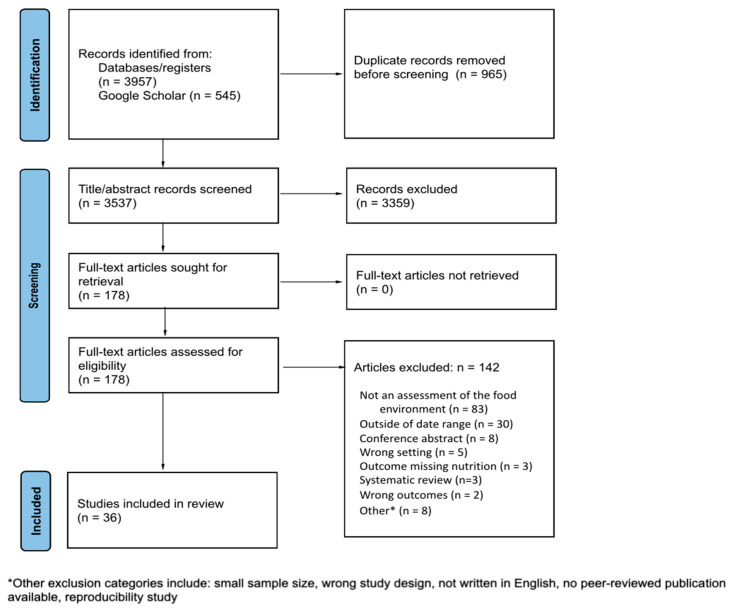
Preferred reporting items for systematic reviews and meta-analyses (PRISMA) flow diagram of identified, screened, and included articles.

The PRISMA flow diagram summarizes the review process (see Figure 2). The database, registry, and Google Scholar searches yielded 4502 results. After removing duplicates, 3537 titles/abstracts were screened for inclusion using Covidence [36]. We requested, obtained, and screened 178 full-text articles for eligibility. The most common reason for excluding an article was that it was not an assessment of the food environment (n = 83). Thirty-six articles met the inclusion criteria for this review.

### 3.1. Description of Included Studies

A summary of the included study characteristics, including assessment type, focus, and scope, is shown in Table 1. Eighteen studies were conducted in the United States [6,7,8,9,10,13,17,18,24,30,31,32,33,34,38,39,40,41], four in Australia [15,28,42,43], three in Brazil [14,27,44], two in Canada [12,45], and one in Egypt [46], Ghana [47], Italy [48], Lebanon [49], New Zealand [50], Norway [51], Spain [11], South Africa [52], and United Arab Emirates [29]. Thirteen studies compared multiple higher education institutions [6,7,8,9,13,15,28,29,38,43,49,50,51]. Ten studies assessed the campus food environment at singular public institutions [10,14,18,24,27,39,40,41,44,47], one at a private institution [34], and twelve studies did not indicate the institution type.

#### 3.1.1. QuADS Results

The authors achieved 83% agreement on item scores before discussing discrepancies and 100% agreement after the consensus process. There was considerable variation in the quality of the included studies, with quality scores ranging from 22% to 97% (See Table 1). The median quality score was 71%, and the average was 68%.

#### 3.1.2. Assessment Characteristics and Psychometric Properties of the Tools Used

Assessment criteria, markers of healthiness, and the psychometric properties of the tools used were extracted for this review. The following assessment criteria were used across the 36 studies: availability (97%), price (64%), food quality (42%), convenience/access (53%), and sustainability practices (14%). Availability refers to the presence or absence of certain types of food or beverages; price refers to the cost of food, comparisons of healthy and unhealthy food, or comparison of prices across venues; convenience/access includes operating hours or distance to a food venue; quality indicates if freshness or visual appearance is inspected; and sustainability includes locally sourced food, organic items, or environmental signage. Seventeen studies (47%) explored additional barriers and facilitators within the campus food environment. Most studies (78%) examined both healthy and unhealthy markers in the university food environment, while eight studies (22%) focused only on healthy items. Indicators of instrument quality included reliability and validity. Seventeen studies explored or reported validity, of which 47% used a validated tool, and 53% used an adapted version of a validated tool. Seventeen studies (47%) described the tool’s reliability (inter-rater or test–retest). Detailed summaries of the specific measures (e.g., price, availability) used within each tool and assessment findings can be found in the articles.

#### 3.1.3. ‘Healthy’ Definitions or Benchmarks Used in Food Environment Assessments

A summary of definitions or benchmarks used to decide the “healthfulness” of a food environment in the studies is provided in Table 2. The definition or benchmark for “healthy” or “healthfulness” was not uniform across all the studies, with each study using a single or combination of dietary guidelines, institutional standards, nutrition labeling regulation, food environment audit tool, or a classification, rating or scoring system which was based on existing nutritional guidelines, regulations, or standards.

Fourteen studies used food environment audit tool criteria like the NEMS criteria [53], the New South Wales criteria [54], and the Health Density Vending Machine Audit Tool (HDVMAT) criteria [55] to determine the healthfulness of a food environment. Nine studies used a classification, index, or rating system based on institutional standards, nutrition policies, guides, or level of food processing. Six studies included in the review employed dietary guidelines or standards as a benchmark, like the US Dietary Guidelines for Americans, the Australian Dietary Guidelines, and the Institute of Medicine recommendations. Three studies used a national or state nutrition labeling regulation or guide. Five studies used no clear standard or criteria, and the definition of “healthy” did not apply to five studies.

### 3.2. Food Environment Tools and Methods

Table 3 presents a count of the assessment tools used across the included studies, where some studies used more than one type of tool to evaluate the campus food environment. The most frequently used assessment tool across the studies included in this review was the NEMS, with eight versions used across various settings (e.g., campus dining, vending). For example, four studies used NEMS for restaurants to assess on-campus eateries [6,14,38,47]. An in-depth summary of the most frequently used assessment tools is described in the following sections.

#### 3.2.1. Nutrition Environment Measures Survey (NEMS) (n = 12) [6,8,9,10,12,14,17,33,38,41,45,47]

The Nutrition Environment Measures Survey (NEMS) was this review’s most frequently used tool across 12 studies, with some studies employing multiple versions of the tool. NEMS is an audit tool used to assess the availability and quality of food options within various food environments. NEMS was initially developed and tested in 2004 and disseminated in 2007 [56]. Since then, NEMS has evolved to include observational measures widely used to audit food outlets, such as restaurants, stores, and vending machines [53,56]. NEMS tools have been used for surveillance, examining healthy food accessibility, and evaluating interventions [56]. Although NEMS measures vary depending on the type of food outlet examined (e.g., stores versus restaurants), they typically assess the availability of healthy foods, barriers, and facilitators to healthy eating, and pricing to produce an overall healthfulness score. For example, the NEMS for vending machines (NEMS-V) provides a score for vending machine product quality [17,53]. The NEMS team also developed a survey to capture an individual’s perception of the food environment, which can be used independently or complement other observational methods [53,56]. Training is required to use a NEMS measure; once trained, individuals record their direct observations of specific food outlets in person or by examining a menu online [53].

#### 3.2.2. Food Environment Quality Index (Food Environment QI) (n = 3) [15,42,50]

The food environment QI was developed to examine the nutritional quality of food offered in the post-secondary setting. It includes three outcome measures (availability, accessibility, and healthy food promotion) and can be used at any campus food outlet, including convenience stores, restaurants, and vending machines. The audit form consists of a list of healthy and unhealthy items within ten categories (e.g., sugary beverages and high-energy snacks). It assigns a point value depending on the availability, accessibility, and promotion of a healthy or unhealthy item within the audited food outlet. Each food outlet is given a composite score and scores for availability, accessibility, and promotion. Food outlets are then classified as unhealthy, intermediate, or healthy.

#### 3.2.3. Byrd-Bredbenner Method (n = 2) [13,29]

Byrd-Bredbenner et al. developed a protocol for examining vending machines at eleven post-secondary campuses [13]. First, data collectors documented information on the food and beverages found in a sample of vending machines. Once collected, data were sent to a central location for analysis. Nutritional quality scores, Nutrients to Maximize Scores, Nutrients to Minimize Scores, and Snack or Beverage Quality Scores were computed for each item and campus. The research team also looked at the percentage of vending machine items that meet healthful criteria for fiber, sugar, fat, saturated fat, sodium, and calories using information from the USDA, nutrition labeling regulations, and Institute of Medicine recommendations as a benchmark.

#### 3.2.4. Full Restaurant Evaluation Supporting a Healthy Dining Environment Audit (FRESH) (n = 2) [7,41]

FRESH was developed to assess varied dining environments (e.g., sit-down, fast-food, or delivery restaurants) by examining the healthfulness of food offerings and environmental supports for healthy food decision-making [7]. The validated audit tool contains 30 items, and a total score was calculated. It is recommended that an individual completes web-based training and practices using the tool, which takes approximately three hours. Each audit requires approximately 20–35 min to complete. FRESH can provide audit results to be used as a baseline, track changes, or compare with other institutions.

#### 3.2.5. Other Measures (n = 17)

All other measures used by the articles included in this systematic review were single-use validated tools, non-validated tools, or tools we could not evaluate. For example, the novel Uni-Food tool was used to comprehensively assess college food environments’ healthiness, equity, and environmental sustainability [28]. The tool consists of 3 components with 68 indicators across 16 domains that can be used to determine if universities are meeting best practice guidelines. A unique feature of this tool is the addition of equity and environmental sustainability since most tools focus exclusively on how food environments impact human health [28].

Salari Bortolot et al. (2019) used a modified version of a validated checklist to examine the availability, quality, and promotion of fruit and vegetables in on-campus establishments that offered meals. The authors also considered the presentation, preparation method, preparation type, placement of fruit and vegetables within stores (e.g., displays near checkout), quality, physical accessibility, price, and advertising of healthy items [44].

Subjective measures were also employed. Four studies used focus groups with unique question guides to understand students’ and employees’ perspectives of the food environment [24,30,31,32]. One study engaged a small group of international graduate students using qualitative photovoice methods to summarize the campus food environment and surrounding community [34]. Ten studies used a survey to measure perceptions of the food environment [9,24,33,39,40,43,48,49,50,51], with only one study using a validated survey [33].

### 3.3. Institutional-Level Articles

Twenty-one studies reported results of an institutional-level audit: eight audited campus eateries [6,7,12,14,27,38,44,52], four examined vending machines [11,13,17,29], one reviewed food stores [8], and eight had a combination of outlet types [10,15,28,41,42,45,46,47]. Two studies detailed the changes to a university food environment over time [12,27], whereas all other studies described the food environment at one time point. The most commonly used tools for the institutional audits were the NEMS (n = 10), FRESH (n = 2), Food Environment QI (n = 2), and a method developed by Byrd-Bredbenner et al. (n = 2). All other studies used a checklist, direct observation, or novel tool or did not specify the measure used. Eight studies examined the food environment at multiple sites ranging from 2 to 15 campuses [6,7,8,13,15,28,29,38]; all others examined a single campus. Study findings are summarized in Table 3, but no comparisons are reported, given the variety of assessments and institutions. The quality scores of institutional-level articles ranged from 22% [52] to 97% [7]. The median quality score (QUADS) was 75%, and the average score was 71%.

#### 3.3.1. Eateries

Eight studies focused on on-campus eateries, which included restaurants, fast-food outlets, food-court-style cafeterias, dining halls, and snack shops. Typically, dining halls serve students with a meal plan, and all other outlets are open to students, faculty/staff, and the public. Four studies in this category used a variation or modification of the NEMS tool [6,12,14,38]. For instance, Horacek et al. (2013) assessed the dining environment on and around 15 university campuses using a modified version of the NEMS for restaurants (NEMS-R) for off-campus restaurants. The authors also adapted the tool for on-campus dining establishments to create the Nutrition Environment Measures Survey–Campus Dining (NEMS-CD). Another study modified the NEMS-R to create a unique NEMS-university campuses tool (NEMS-UC), which was used at two time points to determine if the healthfulness of eateries changed over time [12].

The remaining four studies used various assessment tools [7,27,44,52], with only one exploring changes over time [27].

#### 3.3.2. Food Stores

One institutional-level audit focused solely on food stores in and near 15 universities [8]. The NEMS for stores (NEMS-S) tool was adapted to examine stores located within the university food environment, including stores off-campus but within a 1.5-mile radius beyond the campus property. The study included on-campus convenience and grocery stores; a convenience section of bookstores; and off-campus stores such as healthy food, drug, and grocery stores.

#### 3.3.3. Vending Machines

Four articles in this review were vending machine audits. Two studies used the methodological approach developed by Byrd-Bredbenner et al. [13,29], one compared the NEMS with another validated tool [17], and one used a unique method to assess snacks and beverages found in on-campus vending machines [11].

Byrd-Bredbenner et al. (2012) developed a tool to examine a sample of machines from 11 US university campuses (2607 snack machines and 1650 beverage machines). Faris et al. (2021) used the same methodology at one university with four campuses in the United Arab Emirates. Both studies collected data from vending machines in high-traffic areas; snacks and beverages were categorized based on their nutritional content, and nutritional quality scores were calculated for each item [13,29].

Sankavaram et al. (2021) assessed the nutritional quality of the most frequently purchased vending options at a large land grant institution in the US using two validated tools, the NEMS-V [53] and the HDVMAT [55] and compared their findings. Both tools categorized foods into different categories of healthfulness (e.g., unhealthy, somewhat healthy), and this study provided a side-by-side comparison of the tool’s strengths and weaknesses [17].

#### 3.3.4. Combination of Food Outlet Types

Eight studies explored multiple food outlet types (e.g., campus eateries and vending machines) in university food environments. NEMS was the most frequently used tool in this category [10,41,45,47]. Mensah et al. (2022) used the NEMS concept to categorize food outlets as NCD-healthy, NCD-intermediate, and NCD-unhealthy at a Ghanaian university, then employed spatial distribution analysis to map the location of the establishments, including food service places and food stores. Another study used two versions of the NEMS (NEMS-S and NEMS-CD) to examine 18 dining venues and 39 food stores on one campus and in the surrounding area [10]. Rivera et al. (2020) used the NEMS-V, FRESH, and Convenience Store Supportive Healthy Environment for Life-Promoting Food (SHELF) [57] tools to assess the vending machines, eateries, and convenience stores located on one public university’s campus.

Two studies that originated in Australia used the food environment quality index (QI) [15,42]. The first article detailed the development of the food environment QI and included audit results from 252 food outlets at seven universities [15]. Shi et al. (2018) examined food outlets and vending machines at a large, urban Australian university using visual inspection and the food environment QI.

To accomplish a more comprehensive assessment of multiple food outlets on campus, five studies in this category used more than one tool [10,41,42,46,47]. Additionally, three tools that could be used to assess more than one food outlet type were identified: the food environment QI [15,42], NEMS grab-and-go (NEMS-GG) [45], and Uni-Tool [28]. Of particular interest was the NEMS-GG tool, which was adapted and validated for use within a Canadian university and used at 15 establishments, including coffee shops, takeaway restaurants, and campus stores [45].

### 3.4. Individual-Level Articles

In our review of the literature assessing the food environment at the individual level, we found six articles that primarily focused on consumers’ subjective perceptions of the food environment, and the majority involved qualitative methods [24,30,31,32,33,34]. The study methods varied: three studies used focus groups [30,31,32], one used a student survey [33], one used photovoice [34], and one used a combination of measures [24]. Several of these studies also assessed individual behaviors (e.g., 24 h dietary recall); however, those findings do not fit the scope of the present review. The sample sizes for studies exploring subjective perceptions of the food environment ranged from 6 students [34] to 180 students [33], with a median of 37 participants. The QuADS quality scores of individual-level articles ranged from 38% [34] to 95% [31]. The median quality score was 70%, and the average score was 65%.

Two studies used qualitative approaches to highlight the unique perceptions of special student populations, such as first-year students at a minority-serving institution [30] and international graduate students [34]. Dhillon et al. (2019) used a 10-item semi-structured interview guide based on the Social Cognitive Theory with a sample of 21 first-year students. Prompts explored students’ perceptions of various factors influencing food choices and preferences and the facilitators and challenges of eating healthy on campus [30]. Using a participatory qualitative approach, Malova et al. (2021) trained six international graduate students in photovoice methods to independently capture images and develop narratives about the importance of food on campus and within the surrounding community. After two months, a facilitated session occurred to review the photos, refine narratives, and identify common themes [34].

Skeleton et al. (2020) conducted a qualitative study on the college nutrition environment in which 28 students participated in focus groups, and five additional students provided key informant interviews as a follow-up to further explore the themes of the focus groups. Guided by the Social Ecological Model, focus group questions touched on individual, interpersonal, organizational, and societal influences on food environments. Grounded theory was used for the analysis to allow for emergent themes [32].

Only one study qualitatively explored the perceptions of non-students. Mann et al. (2021) aimed for a representative sample (n = 37) of university employees during semi-structured focus group sessions to learn more about the facilitators and barriers to healthy food on campus. The 12-item discussion guide was based on the Social Ecological Model and covered questions related to barriers to healthy choices on campus, the presentation of healthy options, and strategies for promoting healthy eating [31].

Mengarelli et al. (2021) conducted a mixed-methods study to understand student perceptions of vending machine offerings. Students (n = 20) completed an 11-item questionnaire of demographics and food preferences from vending machines and then participated in a focus group session. The focus group guide was based on an Ecological Framework regarding individual choices and perceptions of vending machine foods. An iterative analysis with multiple coders was applied to the focus group transcripts [24].

Martin et al. (2022) used the validated NEMS-P tool to assess the perceptions of 180 students living on campus. The NEMS-P uses 49 questions about subjective assessments of the community nutrition environment, home food environment, food shopping behaviors, and eating behaviors [33,53]. The analysis used in this study was multivariate logistic regression.

### 3.5. Articles with Multiple SEM Levels

Nine of the included studies incorporated multiple social-ecological levels into the study design or implementation [9,18,39,40,43,48,49,50,51]. The QuADS quality scores of articles targeting multiple SEM levels ranged from 36% [48] to 82% [9]. The median quality score was 62%, and the average score was 62%.

Three studies examined eateries [18,39,48], two assessed vending machines [43,49], one included food stores [9], and three articles evaluated multiple food outlet types [40,50,51]. The most commonly used approach in this category was a combination of an institutional-level audit and a consumer questionnaire [9,39,43,48,49,50,51]. One study paired an institutional-level audit with sales data [18], while another used a student survey, institutional-level audit, and spatial analysis to assess the campus food environment [40]. Among the audits used, five studies used a food or recipe list [18,39,40,48,51], two used direction observation [43,49], one used the food environment QI [50], and one used NEMS-S [9]. The questionnaires employed also varied, with some studies examining students’ dietary behaviors (beyond the scope of this review) and others focused on the perceptions of the campus food environment [39,40,43,49,51].

## 4. Discussion

The university campus food environment provides a unique opportunity for health decision-making and adopting diet-related health behaviors. This review summarized the various methods and tools used to examine college food environments at an institutional and individual level. In total, 36 studies met the eligibility criteria, with 21 focused on assessments of institutional offerings, 6 on personal experiences and perceptions of the university food environment, and 9 examining a combination of factors.

The most commonly used assessment tool was either a validated or modified NEMS audit tool. At the institutional level, the most widely used assessment measure across ten studies was a variation in the NEMS tool [6,8,10,12,14,17,38,41,45,47], although it was applied differently across studies. In one study, the NEMS tool was adapted and validated for university grab-and-go food outlets at a Canadian university [45]. However, the remaining studies used versions of NEMS that were not specifically validated for application in campus food environments. Instead, we observed that articles used versions of NEMS that best fit their research question (e.g., vending or convenience stores). There were no subjective assessments at the institutional level, which indicated no exploration of the role and influence of decision-makers on the campus food environment.

At the individual level, focus groups were often used to understand perceptions of the campus food environment. Depending on the study methods, recruitment, and time to saturation, this approach may produce non-representative findings of the student population [58]. A potential solution would be to consider diverse recruitment strategies until saturation is reached. The development and testing of a validated focus group questionnaire may also allow researchers to compare experiences across institutions in the same way that NEMS can be applied across different environments. When considering multiple SEM levels, surveys were the most frequently used tool to collect individual-level perceptions of the food environment. A few studies in our review used mixed methods to explore the campus food environment across multiple levels, pairing the observational assessments with consumers’ perceptions, which may add value to understanding the campus food environment from a holistic view.

The definition of a campus food environment varied across the literature. Most studies defined it as food outlets on campus property, although six studies also included venues located off-campus but within a predefined boundary (e.g., one mile from the campus center) [6,7,8,9,10,34]. Unless a university has a policy about living on-campus or purchasing a meal plan, students likely receive sources of nourishment off-campus. More than a third (n = 8, 38%) of the institutional-level studies explored multiple settings within the campus food environment (e.g., vending machines and dining halls). Comprehensive evaluations of the entire campus food environment may be limited due to resources or the broad applicability of methodologies across various tools; however, we found the food environment QI [15,42,50], NEMS-GG [45], and Uni-Food [28] tools to encompass multiple aspects of the campus food environment. Moreover, the Uni-Food tool considered domains for university policies and governance impacting the food environment in the assessment [28].

During this review, we came across three ineligible studies assessing college food environments with a particular focus on the environmental health impacts of dining halls [59,60,61]. Their exploration of greenhouse gas emissions produced by campus dining venues was a unique assessment feature and may be a cost-effective way for universities to measure their carbon footprint. Two recent systematic reviews previously considered individual behavior within the college food environment [22,23]. Although our review did not have a behavioral outcome focus, seven studies using innovative approaches for assessing individual behaviors made it to the final screening stage [62,63,64,65,66,67,68]. These articles were screened out due to not reporting campus food environment assessment results or a lack of a description of the methods for assigning a healthfulness score to menu items captured with sales data. Although their methods did not fit our review objectives, these approaches provide innovative strategies for universities to understand the environmental and behavioral impacts of the campus food environment.

The definition or benchmark used for categorizing a food environment as “healthy” or “healthful” varied appreciably across the studies. The “Healthy” classification or “healthfulness” of a food environment was defined using a single or combination of dietary guidelines, nutrition policies, institutional standards, nutrition environment measures, or a classification or rating system. While all of these benchmarks overlap regarding the policies or institutional standards used in developing the guidelines, measures, criteria, or classification system, we wanted to summarize the conceptualizations of healthfulness across all the studies included in the review. The significant variability across the studies underscores the need for a standardized benchmark to allow for easy comparability of findings across studies.

Our review adds to the literature base by exploring how various assessments are used to evaluate the offerings within the campus food environment. The strengths of this review include unrestricted country of origin or assessment type to allow for a thorough review of the existing literature. Additionally, our research team included a librarian to assist with the search across multiple databases, and there was a minimum of two reviewers through each stage of the review process, with discussion for consensus using a third reviewer as needed. Another strength was the inclusion of articles with methods beyond quantitative environmental audits, as we found value in the studies that explored qualitative perceptions of the food environment.

Despite these rigorous methods, there are some notable limitations of the review to consider. First, the search restrictions to include publications from 2012 to 2022 written in the English language only meant that our review was limited to some extent. Additionally, due to the breadth of the literature yielded from our search, the review process and data extraction took a year to complete, creating a gap in time from review onset to publication. Since we focused on the tools used to assess the food environment, some studies exploring the elements of the food environment (e.g., purchasing behavior) did not fit the scope of this review and were screened out.

## 5. Conclusions

Overall, we summarize the methods of a large number of recent studies in the literature focused on the university food environment, indicating an important area of public health nutrition research. From our synthesis of assessment tools, we observed a lack of validated tools for specific use within the campus food environment, which presents an opportunity for future research studies to improve methodological rigor and the ability to compare findings across institutions. Given the variety of methods being used in practice, we suggest that an expert meeting is convened to plan the development and validation of comprehensive campus food environment assessment tools and methodologies as a next step. There is increased interest in studying the influence of campus food environments, likely due to the high rates of food insecurity reported among college students [69]. Most (86%) of the studies included in our review were published in 2016 or later (see Table 1), and we expected the demand for research in the field to increase.

## Figures and Tables

**Table 1:** Descriptive characteristics and scope of included studies.

Assessment Description:						Assessment Focus:			
Author, Year	QuADS Score	Country	Institution Description	Assessment Type	Assessment Scope	Campus Food Environment (Individual Level Only)	Eateries	Vending	Convenience or Food Stores
**Institutional level**									
Begum, J. & Tettey, N., 2020 [38]	58%	USA	Multi-site	Audit	27 eating locations at 2 universities		X		
Byrd-Bredbenner et al., 2012 [13]	75%	USA	Multi-site	Audit	Snack (2607 slots) and beverage (1650 slots) vending machines in 78 buildings on 11 US university campuses			X	
Faris, M. et al., 2021 [29]	75%	UAE	Multi-site	Audit	55 VMs at four university campuses for a total of 57 types of snacks and beverages			X	
Franco, A. et al., 2020 [27]	50%	Brazil	Public	Audit	All establishments selling food and/or beverages at three time points (N = 15 in 2011, N = 17 in 2012 and N = 25 in 2016)		X		
Horacek, T. et al., 2013 [6]	94%	USA	Multi-site	Audit	68 campus dining venues; 175 restaurants at 15 universities		X		
Horacek, T. et al., 2019 [7]	97%	USA	Multi-site	Audit	362 dining venues at 15 college campuses		X		
Horacek, T. et al., 2013 [8]	86%	USA	Multi-site	Audit	81 stores at 15 institutions within a 1.5-mile radius of the campus center				X
Lasala, C. et al., 2022 [11]	56%	Spain	Unknown	Audit	654 food and beverage items from 14 vending machines on a university campus			X	
Lee, K. 2020 [12]	72%	Canada	Unknown	Audit	5 eateries at two time points (2015 and 2017/2018)		X		
Lo, B. et al., 2016 [45]	97%	Canada	Unknown	Audit	15 grab-and-go establishments		X		X
Mann, D. et al., 2021 [28]	83%	Australia	Multi-site	Audit	Food retail outlets (N = 48) at 3 universities		X	X	
Meko, N.M.L. & Jordaan, E.M., 2016 [52]	22%	South Africa	Unknown	Audit	6 food outlets such as tuck shops, cafeterias, or takeaway food outlets		X		
Mensah, D. et al., 2022 [47]	89%	Ghana	Public	Audit	138 food outlets		X		X
Mohamed, E. et al., 2016 [46]	31%	Egypt	Unknown	Audit	17 food courts		X		X
Pulz, I.S. et al., 2017 [14]	75%	Brazil	Public	Audit	6 restaurants and 13 snack bars		X		
Rivera, B. et al., 2020 [41]	61%	USA	Public	Audit	16 food retail outlets, 14 dining locations, 2 campus convenience stores, 35 vending machines		X	X	X
Roy, R. et al., 2016 [15]	75%	Australia	Multi-site	Audit	252 food outlets across 7 campuses		X	X	X
Salari Bortolot, B. et al., 2019 [44]	69%	Brazil	Public	Audit	8 food establishments that offered meals		X		
Sankavaram, K. et al., 2021 [17]	86%	USA	Unknown	Audit	12 most popular items in the top 20 revenue-generating vending machines on one university campus			X	
Shi, Y. et al., 2018 [42]	64%	Australia	Unknown	Audit	30 food outlets and 62 vending machines		X	X	
Tseng, M. et al., 2016 [10]	78%	USA	Public	Audit	18 campus dining venues and 2 on-campus and 37 off-campus food stores		X		X
**Individual level**									
Dhillon, J. et al., 2019 [30]	72%	USA	Unknown	Focus group	Focus group interviews of 21 first-year students	X			
Malova, E. et al., 2021 [34]	38%	USA	Private	Photovoice	Photovoice assessment of the campus and surrounding communities (N = 6)	X			
Mann, G. et al., 2022 [31]	95%	USA	Unknown	Focus group	8 focus group discussions including 25 faculty and 12 staff from various departments	X			
Martin, S. & McCormack, L. 2022 [33]	44%	USA	Unknown	Student survey	Student survey (NEMS-P): on-campus students (N= 180)	X			
Mengarelli, C.A. et al., 2021 [24]	67%	USA	Public	Focus group, student survey	Focus group interviews and a survey of 20 students on campus			X	
Skelton, K. & Evans, R., 2020 [32]	74%	USA	Unknown	Focus groups and key informant interviews	A non-probability convenience sample of 33 undergraduate college students participated in focus groups (28) and key informant interviews (5)	X			
**Multiple levels**									
Calvez, K. et al., 2016 [40]	62%	USA	Public	Audit, spatial analysis, survey	Survey of 263 students and audit of 17 dining locations		X		X
Carey, G. et al., 2017 [39]	59%	USA	Public	Audit, survey	Pre and post-intervention surveys (N = 200) were administered at 2 food locations where the intervention was implemented		X		
Horacek, T. et al., 2018 [9]	82%	USA	Multi-site	Audit, survey	Audits of convenience food stores at 13 institutions: 27 convenience/drug stores and 14 on-campus stores. Behavioral survey of 1401 students from 13 universities.				X
Leishner, K. et al., 2018 [18]	58%	USA	Public	Audit, sales data	Audit of campus dining food list (662 food items assessed)		X		
Martinez-Perez, N. et al., 2022 [51]	74%	Norway	Multi-site	Audit, survey	Audit of 2 campuses that included 256 foods/drinks at 7 canteens, 3 coffee shops, and 2 vending machines. Survey of 129 students and staff on food purchasing, food choice, and opinion of campus food environment.		X	X	
Ng, K.W. et al., 2019 [43]	67%	Australia	Multi-site	Audit, survey	Audit of vending machines (1259 slots in 49 machines) and survey (222 university staff and students) at five campuses			X	
Rahi, B. et al., 2022 [49]	51%	Lebanon	Multi-site	Audit, survey	Audit of 21 vending machines across 8 university campuses and student surveys (N = 603)			X	
Roy, R. et al., 2019 (67%) [50]	67%	New Zealand	Multi-site	Audit, survey	Audit of 57 food outlets across 6 university campuses and student survey (N = 1954)		X	X	X
Turconi, G. et al., 2012 [48]	36%	Italy	Unknown	Audit, survey	Audit of 216 meals served in one cafeteria and survey (374 university students and employees)		X		

**Table 2:** Healthfulness definitions or benchmarks used in included studies *.

Healthy Definition	Count	Author, Year
Food Environment Audit Tool Criteria	14	Begum et al., 2020 [38]; Horacek et al., 2013 [6]; Horacek et al., 2019 [7]; Horacek et al., 2013 [8]; Horacek et al., 2018 [9]; Lee et al., 2016 [12]; Lo et al., 2016 [45]; Mensah et al., 2022 [47]; Ng et al., 2019 [43]; Pulz et al., 2017 [14]; Rivera et al., 2020 [41]; Roy et al., 2016 [15]; Sankavaram et al., 2021 [17]; Tseng et al., 2016 [10]
Classification, index, rating, or scoring system	9	Begum et al., 2020 [38]; Byrd-Bredbenner et al., 2012 [13]; Calvez et al., 2016 [40]; Carey et al., 2017 [39]; Faris et al., 2021 [29]; Martinez-Perez et al., 2022 [51]; Mensah et al., 2022 [47]; Roy et al., 2019 [50]; Turconi et al., 2012 [48]
Dietary guidelines or institutional standards	6	Byrd-Bredbenner et al., 2012 [13]; Leishner et al., 2018 [18]; Mann et al., 2021 [28]; Martinez-Perez et al., 2022 [51]; Salari et al., 2019 [44]; Shi et al., 2018 [42]
Not applicable	5	Dhillon et al., 2019 [30]; Malova et al., 2021 [34]; Mann et al., 2021 [31]; Mengarelli et al., 2021 [24]; Skelton & Evans, 2020 [32]
Unspecified benchmark	4	Franco et al., 2020 [27]; Martin et al., 2022 [33]; Meko et al., 2016 [52]; Mohamed et al., 2016 [46]
Nutrition labeling regulation or guide	3	Byrd-Bredbenner et al., 2012 [13]; Lasala et al., 2022 [11]; Rahi et al., 2022 [49]

* Note: some studies used multiple benchmarks.

**Table 3:** Count of assessment tools identified in included studies *.

		SEM Level Assessed		
Tool	Count	Institutional	Individual	Multiple
Nutrition Environment Measures Survey (NEMS)	17			
* NEMS-Restaurants (n = 4)*		Begum, J. & Tettey, N., 2020 [38], Horacek, T. et al., 2013 [6], Mensah, D. et al., 2022 [47], Pulz, I.S. et al., 2017 [14]		
* NEMS-Stores (n = 4)*		Horacek, T. et al., 2013 [8], Mensah, D. et al., 2022 [47], Tseng, M. et al., 2016 [10]		Horacek, T. et al., 2018 [9]
* NEMS-Campus Dining (n = 2)*		Horacek, T. et al., 2013 [6], Tseng, M. et al., 2016 [10]		
* NEMS-Grab-and-Go (n = 2)*		Begum, J. & Tettey, N., 2020 [38], Lo, B. et al., 2016 [45]		
* NEMS-Vending (n = 2)*		Rivera, B. et al., 2020 [41], Sankavaram, K. et al., 2021 [17]		
* NEMS-Corner Stores (n = 1)*		Begum, J. & Tettey, N., 2020 [38]		
* NEMS-Perceived (n = 1)*			Martin, S. & McCormack, L. 2022 [33]	
* NEMS-University Campuses (n = 1)*		Lee, K. 2020 [12]		
Non-Validated Survey	9		Mengarelli, C.A. et al., 2021 [24]	Calvez, K. et al., 2016 [40], Carey, G. et al., 2017 [39], Horacek, T. et al., 2018 [9], Martinez-Perez, N. et al., 2022 [51], Ng, K.W. et al., 2019 [43], Rahi, B. et al., 2022 [49], Roy, R. et al., 2019 [50], Turconi, G. et al., 2012 [48]
Food or Recipe List	6	Shi, Y. et al., 2018 [42]		Calvez, K. et al., 2016 [40], Carey, G. et al., 2017 [39], Leishner, K. et al., 2018 [18], Martinez-Perez, N. et al., 2022 [51], Turconi, G. et al., 2012 [48]
Direct Observation	4	Byrd-Bredbenner et al., 2012 [13], Shi, Y. et al., 2018 [42]		Ng, K.W. et al., 2019 [43], Rahi, B. et al., 2022 [49]
Focus Group/Interview Guide	4		Dhillon, J. et al., 2019 [30], Mann, G. et al., 2022 [31], Mengarelli, C.A. et al., 2021 [24], Skelton, K. & Evans, R., 2020 [32]	
Photographs	4	Faris, M. et al., 2021 [29], Lasala, C. et al., 2022 [11]	Malova, E. et al., 2021 [34]	Ng, K.W. et al., 2019 [43]
Food Environment Quality Index	3	Roy, R. et al., 2016 [15], Shi, Y. et al., 2018 [42]		Roy, R. et al., 2019 [50]
Full Restaurant Evaluation Supporting a Healthy Dining Environment (FRESH)	2	Horacek, T. et al., 2019 [7], Rivera, B. et al., 2020 [41]		
Geographic Information System Mapping	2	Mensah, D. et al., 2022 [47]		Calvez, K. et al., 2016 [40]
Validated Checklist	2	Franco, A. et al., 2020 [27], Salari Bortolot, B. et al., 2019 [44]		
Health Density Vending Machine Audit Tool (HDVMAT)	1	Sankavaram, K. et al., 2021 [17]		
Observational Checklist	1	Mohamed, E. et al., 2016 [46]		
Original Qualitative Instrument	1	Pulz, I.S. et al., 2017 [14]		
Convenience Store Supportive Healthy Environment for Life-Promoting Food (SHELF)	1	Rivera, B. et al., 2020 [41]		
Structured Questionnaire	1	Mohamed, E. et al., 2016 [46]		
Uni Food	1	Mann, D. et al., 2021 [28]		
Unspecified	1	Meko, N.M.L. & Jordaan, E.M., 2016 [52]		

* Note: Many studies utilized multiple tools to assess their university food environments. Studies varied in the application of the assessment tools across SEM levels. See Table 1 for details on the study assessment application.

## Data Availability

The original contributions presented in the study are included in the article/Supplementary Material; further inquiries can be directed to the corresponding author.

## Supplementary Materials

The following supporting information can be downloaded at: https://www.mdpi.com/article/10.3390/nu16101426/s1, Supplementary File S1: Database search terms.
