# Supplementary material for: Assessing the Healthfulness of University Food Environments: A Systematic Review of Methods and Tools
Source: Nutrients. 2024 May 9;16(10):1426. doi: 10.3390/nu16101426 (PMC11123862; doi:10.3390/nu16101426)
Supplement: Supplementary file 1 [file nutrients-16-01426-s001.zip › nutrients-2991081-supplementary.pdf]

Supplementary File S1. Search strategy for the databases included in the review (* indicates truncation to broaden the search results to include various word endings)

Number	Database	Search terms	Number of records (As of November, 2022)
#1	**PubMed & Cochrane Central**	“eating habit” or “eating habits” or “eating behavior” or “eating behaviors” or “eating behaviour” or “eating behaviours” or “purchasing behavior” or “purchasing behaviors” or “purchasing behaviour” or “purchasing behaviours” or “food choice” or “food choices” or “meal behavior” or “meal behaviors” or “meal behaviour” or “meal behaviours” or Food Preferences [MeSH]<br>AND “Food environment” or “food environments” or “calorie posting” or “calorie postings” or “caloric posting” or “caloric postings” or “calorie information” or “caloric information” or “nutritional information” or “nutrition information” or “beverage tax” or “beverage taxes” or “point of purchase item” or “point of purchase items” or “meal plan” or “meal plans” or Nutrition Policy [MeSH] or “food pantry” or “food pantries” or “food access” or “food accessibility” or dining<br>AND Universities [MeSH] or college* or post secondary or post-secondary or postsecondary or universit*	1999
#2	**Web of Science & ProQuest Nursing and Allied Health**	“Eating Habit*” or “eating behav*” or “purchasing behav*” or “food choice*” or “meal behav*” or “food preference*”<br>AND<br>“food environment*” or “calor* posting*” or “calor* information” or “nutrition* information” or “beverage tax*” or “point of purchase item*” or “meal plan*” or “nutrition* polic*” or “food pantr*” or “food access*” or “food preference*” or dining<br>AND<br>universit* or colleg* or post-secondary or postsecondary or post secondary	392
#3	**ProQuest Nursing and Allied Health**	Abstract(“Eating Habit*” or “eating behav*” or “purchasing behav*” or “food choice*” or “meal behav*” or “food preference*”) or title (“Eating Habit*” or “eating behav*” or “purchasing behav*” or “food choice*” or “meal behav*” or “food preference*”) AND abstract (“food environment*” or “calor* posting*” or “calor* information” or “nutrition* information” or “beverage tax*” or “point of purchase item*” or “meal plan*” or “nutrition* polic*” or “food pantr*” or “food access*” or “food preference*” or dining) or title (“food environment*” or “calor* posting*” or “calor* information” or “nutrition* information” or “beverage tax*” or “point of purchase item*” or “meal plan*” or “nutrition* polic*” or “food pantr*” or “food access*” or “food preference*” or dining) AND<br>abstract (universit* or colleg* or post-secondary or postsecondary or post secondary) or title (universit* or colleg* or post-secondary or postsecondary or post secondary)	125
#4	**APA PsycInfo**	DE “Eating Behavior+” or “eating Habit*” or “eating behav*” or “purchasing behav*” or “food choice*” or “meal behav*” or DE “Food preferences” or “food preference*” AND “food environment*” or “calor* posting*” or “calor* information” or “nutrition* information” or “beverage tax*” or “point of purchase item*” or “meal plan*” or “nutrition* polic*” or “food pantr*” or “food access*” or dining<br>DE “Colleges+” or college* or universit* or post-secondary or postsecondary or post secondary	1239

#5	**CINAHL Complete**	MH “Eating Behavior+” or MH “Food Habits” or “eating behav*” or “eating habit*” or “purchasing behav*” or ME “Food Preferences” or “food preference*” or “food choice*” or “meal behav*” AND<br>“Food environment*” or “calor* posting*” or “calor* information” or “nutrition* information” or “beverage tax* or “point of purchase item*” or “meal plan*” or MH “Nutrition Policy+” or “nutrition* polic*” or “food pantr*” or “food access*” or dining ANDMH “Colleges and universities+” or college* or universit* or postsecondary or post-secondary or post secondary	202
#6	**Google Scholar**	"campus food environment" and "assessment" AND<br>"university food environment" and "assessment" AND<br>"college food environment" and "assessment" AND<br>"assessment" and "college" and "food environment"	545 (as of July 6, 2023)
Total	#1 AND #2 AND #3 AND #4 AND #5 AND #6		**4502**
